# Effect of Angular Velocity on Sensors Based on Morphology Dependent Resonances

**DOI:** 10.3390/s140407041

**Published:** 2014-04-22

**Authors:** Amir R. Ali, Tindaro Ioppolo

**Affiliations:** 1 Mechanical Engineering Department, Micro-Sensor Laboratory, Southern Methodist University, 3101 Dyer Street, Dallas, TX 75205, USA; E-Mail: arahmed@smu.edu; 2 Mechanical Engineering Department, Microsystems Research Laboratory, Southern Methodist University, 3101 Dyer Street, Dallas, TX 75205, USA

**Keywords:** angular velocity, spherical resonator, morphology dependent resonances (MDR), polydimethylsiloxane (PDMS), polymeric sensor, fiber optics, sensor fiber

## Abstract

We carried out an analysis to investigate the morphology dependent optical resonances shift (MDR) of a rotating spherical resonator. The spinning resonator experiences an elastic deformation due to the centrifugal force acting on it, leading to a shift in its MDR. Experiments are also carried out to demonstrate the MDR shifts of a spinning polydimethylsiloxane (PDMS) microsphere. The experimental results agree well with the analytical prediction. These studies demonstrated that spinning sensor based on MDR may experience sufficient shift in the optical resonances, therefore interfering with its desirable operational sensor design. Also the results show that angular velocity sensors could be designed using this principle.

## 1. Introduction

In recent years, high optical quality factor resonators have been used for many applications. Some of these include those in optical telecommunication [1–3] biological [4] and mechanical sensing [5–16]. References [17–19] describe a detailed review of recent MDR applications. The MDR are optical modes that are observed in dielectric resonator, and are excited by coupling light from a tunable laser into the resonator using a single mode optical fiber.

**Figure 1.**
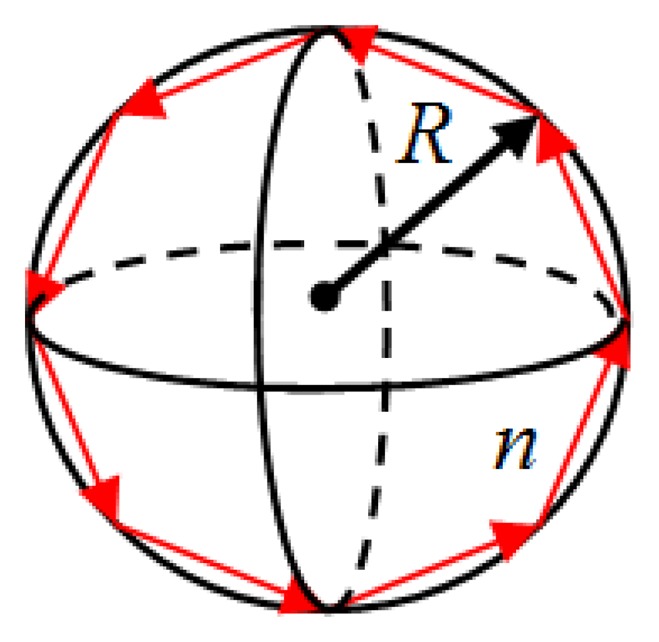
Ray optics description of MDR in a sphere.

A simplified description of the MDR phenomenon can be obtained by using geometric optics as shown in Figure 1. This description is valid when the wavelength of the light used to excite the optical modes is much smaller than the size of the optical cavity. In this geometric view, light coupled into the microsphere (for example using a single mode optical fiber) circles the interior of the sphere through total internal reflection as long as the refractive index of the sphere is larger than that of the surrounding medium.

The condition for optical resonance is 2*π Rn* = *lλ*, where *λ* is the vacuum wavelength of the light (supplied by a laser), *l* is an integer, *R* is the sphere radius, and *n* is the sphere refractive index. An external effect applied to the sphere that induces a change in both the radius, *ΔR*, (mechanical strain) and the refractive index, *Δn*, (due to mechanical stress) leads to a shift in the optical resonance (MDR) as follows:

(1) $$\frac{\Delta R}{R}+\frac{\Delta n}{n}=\frac{\Delta \lambda }{\lambda }$$

**Figure 2.**
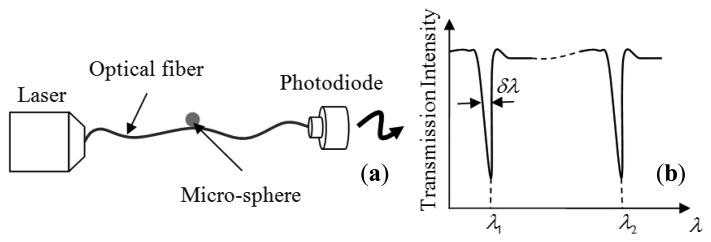
(**a**) Schematic of sensor system and (**b**) observed transmission spectrum.

Therefore, any change in the index of refraction and radius of the microsphere induced by the external effect can be sensed by monitoring the change (shift) in the resonance (MDR) of the microsphere. Our earlier studies on MDR have shown that for most sphere materials (silica and polymers), *ΔR*/*R* dominates over *Δn*/*n* and the latter can be neglected [7]. The general optical arrangement for these sensors is depicted in Figure 2. The optical modes are excited by coupling light from a tunable laser (with nominal power of a few mW) into the sphere using a single mode optical fiber as shown in Figure 2a. The optical fiber which carries light from the tunable laser serves as an input/output port for the microsphere. When the microsphere is brought in contact with a tapered section of the optical fiber its optical resonances are observed as sharp dips in the transmission spectrum at the end of the fiber as illustrated in Figure 2b.

A key factor that makes this phenomenon attractive for sensor applications is the very large optical quality factors, *Q*, of the optical resonances. The observed line-width, *δλ*, is related to the optical quality factor as *Q* = *λ*/*δλ*. In our laboratories we can routinely achieve optical quality factor of 10^7^.

Here, we investigate the effect of angular velocity on the MDR shifts of spherical resonators that are used as sensing element as described above. The elastic deformation that is induced in a spinning resonator due to the centrifugal force, may lead to a sufficient shift in the optical resonances and therefore interfering with its desirable operational sensor design. Also this principle could be used for the development of angular photonic sensors. Note that the shift induced in the optical modes by the centrifugal force should not be confused with the Sagnac effect, since the latter requires the interference of two beams traveling in two opposite directions [15].

**Figure 3.**
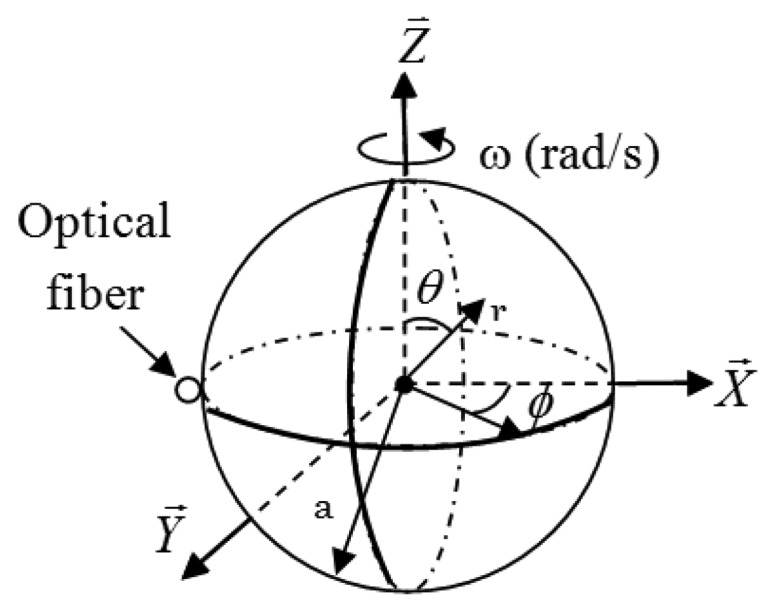
Geometry of a rotating sphere.

When a microsphere (sensing element) of radius *a*, and index of refraction *n* is rotating with an angular velocity *ω* (see Figure 3), its morphology (shape and index of refraction) is perturbed due to the centrifugal force acting on the resonator. This in turn induces a shift in its optical resonances as described in Equation (1).

### Analysis

An analytical expression for the MDR shifts induced by the angular velocity *ω* is obtained by solving the Navier equation of linear elasticity:

(2) $$\nabla^{2}u+\frac{1}{1-2v}\nabla (\nabla \cdot u)+\frac{f}{G}=0$$

Here *u* is the displacement of a point within the sphere, *G* is the shear modulus, *v* is the Poisson ratio and *f* is the body force (centrifugal force) acting on the rotating sphere. The boundary conditions to Equation (2) for the rotating sphere are:

(3) $$\sigma_{rr}=0\;\text{and}\;\sigma_{r\theta }=0\;\text{at}\;r=a$$

where *σ_rr_* and *σ_rθ_* are the normal and tangential components of stress. The centrifugal force acting on the sphere can be written in terms of its radial and tangential component as follows:

(4) $$f=\rho \omega^{2}r\sin\theta \left(\sin\theta \vec{r}+\cos\theta \vec{\theta }\right)$$

Here *ρ* is the density of the sphere, *r⃗* and *θ⃗* are unit vectors (see Figure 3).

The solution to Equation (2) is the sum of the homogenous and the particular solution. The particular solution for the radial component of the displacement of a point on the surface of the sphere can be written as [8,20]:

(5) $$u_{r}^{p}=\frac{a^{3}(-7+20P_{2}(\cos\theta ))(-1+2v)\rho \omega^{2}}{420G(-1+v)}$$

where *P_2_*(*cosθ*) is the Legendre polynomial of degree two of the first kind.

The homogenous solution for the radial component of the displacement of a point on the surface of the sphere can be express as follows [8,19]:

(6) $$u_{r}^{h}=\frac{-\rho \omega^{2}a^{3}}{G}\left[\frac{\left(-3+v)(-1+2v\right)}{60(-1+v^{2})}+\frac{\left(-21+v(16+17v))P_{2}(\cos\theta \right)}{21(-1+v)(7+5v)}\right]$$

More details regarding the above analysis Equations (5) and (6) can be found in [8]. The total radial component of the displacement of a point on the surface of the sphere is obtained by combining Equations (5) and (6). The relative change in the sphere radius on the plane *θ* = *π*/2 (plane where the light is travelling inside the sphere) can be simplified using Equations (5) and (6) as:

(7) $$\frac{\Delta \lambda }{\lambda }=\frac{\Delta a}{a}=\frac{a^{2}(17+(6-5v)v)\rho \omega^{2}}{30G(1+v)(7+5v)}$$

**Figure 4.**
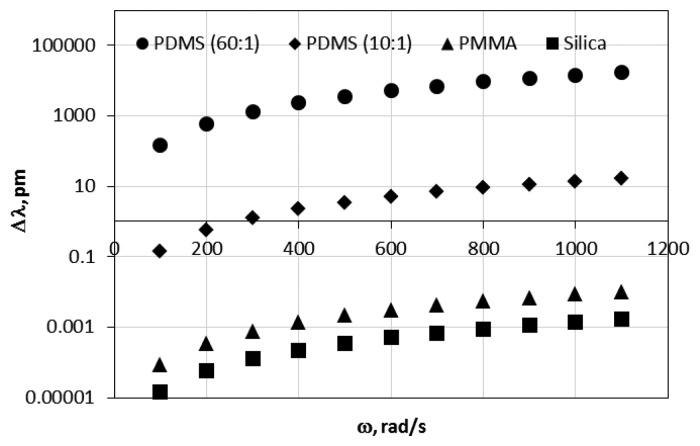
MDR shift using Equation (7) for silica, PMMA and PDMS 10:1 and 60:1 resonators.

As was expected the MDR shift is directly proportional to the sphere density and also is a quadratic function of the sphere radius and the angular velocity. Figure 4 shows the MDR shifts as a function of the angular velocity for a silica (G = 3 × 10^10^ GPa, *ν* = 0.17), polymethylmethacrylate (PMMA, G = 2.6 × 10^9^ GPa, *ν* = 0.35) polydimethylsiloxane (PDMS 10:1, 10 parts of polymeric base and one part of curing agent by volume, with G = 300 kPa, *ν* = 0.49) and (PDMS 60:1, 60 parts of polymeric base and one part of curing agent, by volume with G = 1 kPa, *ν* = 0.49) with a radius of the resonator of 500 μm. As shown in Figure 4, resonators with smaller shear modulus *G* experience larger MDR shift due to the larger mechanical deformation induced by the applied external angular velocity. Figure 4 also shows that the MDR shifts that are induced in a spinning resonator are very small for silica and PMMA resonators in the range of the calculated angular velocity.

## 2. Experimental Section

**Figure 5.**
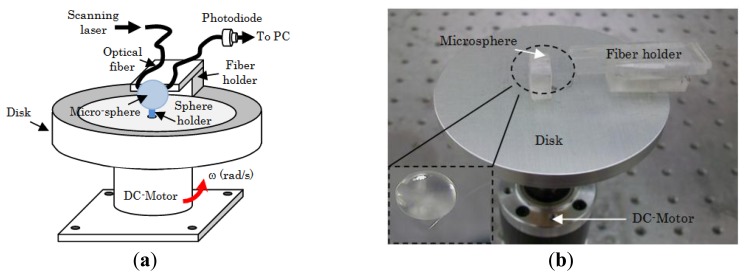
(**a**) Schematic and (**b**) photograph of the experimental setup.

We carried out a series of experiments to investigate the effect of angular velocity on the MDR shifts of 60:1 and 10:1 PDMS. The opto-electronic setup that we used to excite and monitor the MDR is the same as the one described in [7]. Briefly, the output of a distributed feedback (DFB) laser (nominal central wavelength 1.312 μm) was coupled at one end of a single mode optical fiber, while the other end was terminated to a photodiode to monitor the transmission spectrum as shown in Figure 2. The DFB laser was current-tuned to excite the optical resonances. The light was coupled evanescently into the microsphere using a tapered section of a single mode optical fiber. The optical fiber was brought in contact with the resonator using a micro translation stage. Once resonances were observed trough the transmission spectrum, the fiber holder was glued to the disk (see Figure 5). Note that the coupling was obtained while the disk was not rotating, and also for this experimental configuration the DFB laser and the photodiode were not rotating with the disk. This configuration may lead to a fragile coupling between the sphere and the fiber; however the system can be made more robust using a planar wave guide coupler as reported in [21]. Also the tapered section of the optical fiber was firmly attached to the resonator due to the softness of the PDMS. This allowed a stable coupling between the sphere and the optical fiber during the experiments. The microsphere was fabricated as follows. The PDMS base (Sylgard 184 by Dow Corning, Midland, MI, USA) is mixed with the curing agent (60:1 or 10:1 ratio by volume). Next a 125 μm-diameter silica fiber is dipped into the mixture to form a sphere at the fiber's tip and then cured for 4 hours at a temperature of 100 °C. This simple technique allows the fabrication of high optical quality factor microspheres (see Figure 7 below). The silica stem is used to attach the microsphere to the rotating disk. Figure 5 shows a schematic and a photograph of the experimental setup.

The microsphere was mounted on a metallic disk and attached to it using the silica stem. The metallic disk was mechanically coupled to a DC motor (see Figure 5). The angular velocity of the disk was changed by varying the voltage that was supplied to the DC motor. The MDR shifts were recorded on a PC. During the experiments the temperature was kept constant. The entire experimental setup was mounted on a floating optical table to reduce noise induced by vibrations.

## 3. Results and Discussion

**Figure 6.**
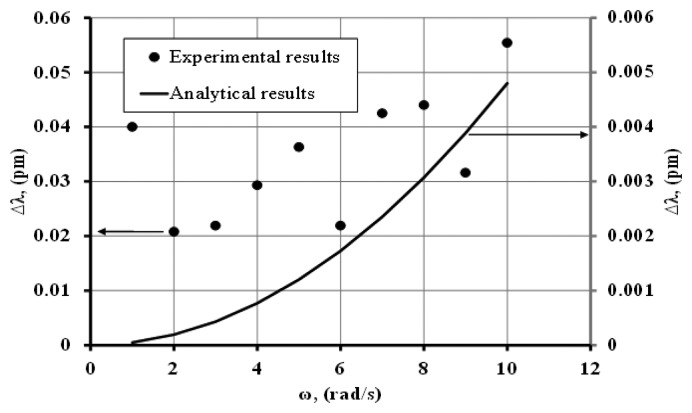
Comparison of experimental results with analytical prediction for a (10:1) PDMS microsphere.

Figure 6 shows the experimental and analytical results predicted by Equation (7) for a microsphere made of (10:1) PDMS with a radius of 500 μm. The optical quality for this resonator was ∼10^7^. As depicted in Figure 6 the (10:1) PDMS microsphere did not show any measurable MDR shift as demonstrated from the scattering of the data points (see Figure 6). Therefore the scatter in the data is an indication of the noise floor (∼0.04 pm) during a typical measurement with *ω* ≠ 0, that could be caused by vibration, temperature, and coupling fluctuations between the sphere and the tapered section of the optical fiber.

**Figure 7.**
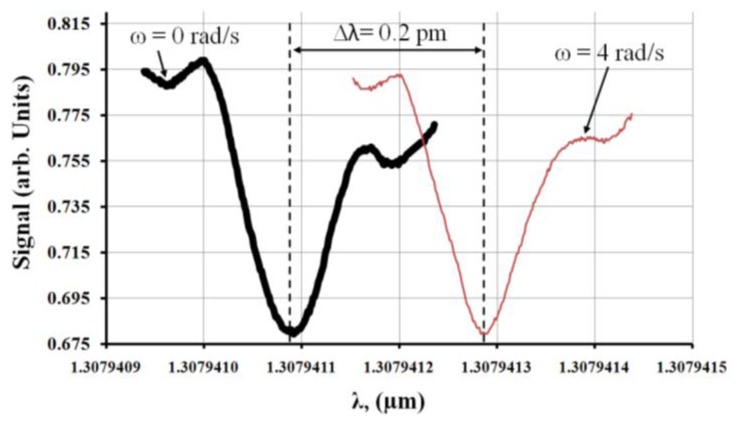
Transmission spectrum of MDR for a (60:1) PDMS microsphere.

Figure 7 shows the transmission spectrum of the observed MDR shift with *ω* = 0 rad/s and *ω* = 4 rad/s for a (60:1) PDMS microsphere. For this experiment the observed optical quality factor was ∼10^7^.

**Figure 8.**
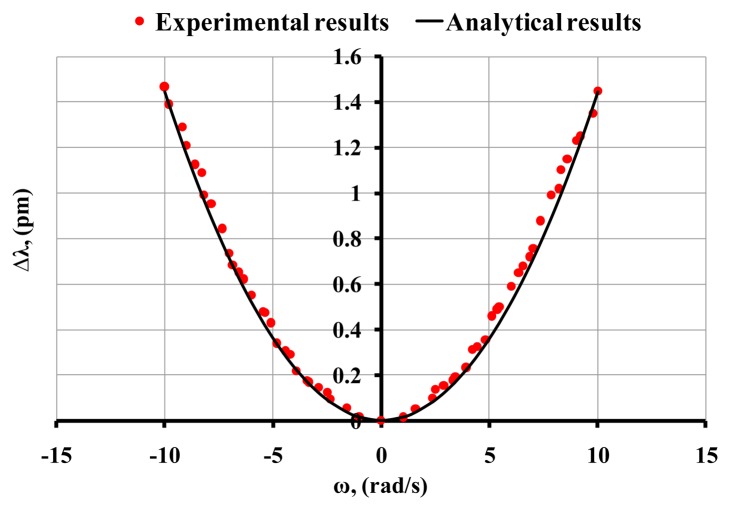
Comparison of experimental results with analytical prediction for a (60:1) PDMS resonator.

Figure 8 shows the MDR shifts for a (60:1) PDMS microsphere for a range of angular velocity between −10 and 10 rad/s. As shown in Figure 8 the experimental results agree reasonably well with the analytical results predicted by Equation (7). For the analytical results we used G = 1,000 kPa and *ν* = 0.49. During the experiments the observed optical quality factor was ∼10^7^. A comparison of Figures 6 and 8 (hard and soft PDMS resonator) leads to the conclusion that the observed optical shifts are due to the elastic deformation that is induced by imposing an external angular velocity since in the case of 10:1 PDMS (hard material compared to the 60:1 PDMS) no measurable shift was obtained.

## 4. Conclusions

The results show that polymeric resonators may experience MDR shift when exposed to an external angular velocity. The analytical results agree reasonable well with the experimental results. Recently soft polymers have been used as materials for the fabrication of optical resonators. Therefore, the optical shift induced in these resonators by the angular velocity can interfere with its desirable operational sensor design. These results are also the first step towards the design and development of angular velocity sensors that are based on this principle.
